# Long-term outcomes according to additional treatments after endoscopic resection for rectal small neuroendocrine tumors

**DOI:** 10.1038/s41598-019-40668-6

**Published:** 2019-03-20

**Authors:** Jae Hwang Cha, Da Hyun Jung, Jie-Hyun Kim, Young Hoon Youn, Hyojin Park, Jae Jun Park, Yoo Jin Um, Soo Jung Park, Jae Hee Cheon, Tae Il Kim, Won Ho Kim, Hyun Jung Lee

**Affiliations:** 1 Department of Internal Medicine, Gangnam Severance Hospital, Yonsei University College of Medicine, Seoul, 06273 Korea; 2 Department of Internal Medicine, Institute of Gastroenterology, Yonsei University College of Medicine, Seoul, 03722 Korea; 3 Department of Internal Medicine and Liver Research Institute, Seoul National University College of Medicine, Seoul, 03080 Korea; 4 Department of Internal Medicine, Dong-A University College of Medicine, Busan, 49201 Korea; 5 Department of Internal Medicine, CHA Bundang Medical Center, CHA University, Seongnam, 13496 Korea

## Abstract

The present study aimed to investigate treatment strategies determining additional treatment after endoscopic resection (ER) of rectal neuroendocrine tumor (NET)s and long-term outcomes of endoscopically resected rectal NETs. We analyzed a total of 322 patients medical records of patients who underwent ER for rectal NETs. Rectal NETs initially resected as polyps and treated with conventional endoscopic mucosal resection (EMR) were observed more frequently in the non-curative group (*P* = 0.041 and *P* = 0.012, respectively). After ER, only 44 of the 142 patients (31.0%) who did not meet the criteria for curative resection received additional salvage treatment. In multivariate analysis, lesions diagnosed via biopsies (OR, 0.096; *P* = 0.002) or suspected as NETs initially (OR, 0.04; *P* = 0.001) were less likely to undergo additional treatment. Positive lymphovascular invasion (OR 61.971; *P* < 0.001), positive (OR 75.993; *P* < 0.001), or indeterminate (OR 13.203; *P* = 0.001) resection margins were more likely to undergo additional treatment. Although lymph node metastasis was found in 6 patients, none experienced local or metastatic tumor recurrence during the median follow-up of 40.49 months. Long-term outcomes after ER for rectal NETs were excellent. The prognosis showed favorable outcomes regardless of whether patients receive additional salvage treatments.

## Introduction

Neuroendocrine tumors (NETs) are rare malignancies that arise from the neuroendocrine cells^1^; their clinical behavior and prognosis are highly heterogenous and depend largely on their anatomic location^2^. Rectal NETs represent approximately one-third of all gastrointestinal tract NETs; they are the most common after small bowel NETs^3^. The incidence of rectal NETs appears to be increasing because of the widespread use of screening endoscopy for detecting colorectal cancers. The US-based Surveillance, Epidemiology, and End results registry database reveals that the age-adjusted incidence of rectal NETs have shown an approximately 10-fold increase in the past 35 years^4^. Because more than 50% of rectal NETs are diagnosed incidentally on endoscopy, the majority of them (80–88%) remain localized and less than 10 mm in size at diagnosis^5,6^. This translates into better long-term outcomes than NETs at other sites with an overall 5-year survival rate of 74–88%^3^.

The most important factor in treatment strategy – whether to perform endoscopic resection (ER) or radical surgery for NETs – is the risk of lymph node (LN) metastasis. Considering the aforementioned characteristics, most rectal NETs are treated with ER. There have been various studies regarding the risk factors for LN metastasis to identify high-risk patients, mainly to prevent unfavorable outcomes and decrease the incidence of unnecessary surgeries. The predictive factors include tumor size, depth of invasion, lymphatic invasion, resection margin, mitotic rate, and Ki-67 index^7–9^. After ER, current guidelines recommend additional salvage therapy for high-risk rectal NETs^10,11^.

However, the prognostic value of risk factors associated with the presence of LN metastasis varies between studies. There is insufficient evidence with regard to long-term prognosis after ER and which patients might benefit from additional treatment. Sekiguchi *et al*.^12^ reported that no recurrence was detected in patients with endoscopically treated rectal NETs despite the presence of lymphovascular invasion (LVI) in nearly 50% of the lesions; Nakamura *et al*.^13^ showed excellent prognosis even with a low curative resection rate (65.3%). Therefore, the present study aimed to investigate treatment strategies determining additional treatment after ER of rectal NETs and long-term outcomes according to additional salvage treatments performed in endoscopically resected rectal NETs.

## Results

### Patient demographic and clinical characteristics

A total of 322 patients were included in this study. Table 1 shows demographic and clinical characteristics of patients with endoscopically resected rectal NETs. In the 322 patients, the mean age at diagnosis was 47.67 ± 11.47 years, and 207 (64.3%) were men. Nearly half of the lesions were not suspected as NETs initially (diagnosed via biopsy in 102 (31.7%) patients and resected as polyps and then diagnosed in 30 (9.3%)). Prior to ER, endoscopic ultrasonography was performed in 44 (13.7%) patients, whereas abdominal imaging, including abdominal US or abdominal CT, were done in most patients (309 patients, 96.0%). Lesions were treated with conventional EMR, modified EMR, and ESD in 97 (30.1%), 122 (37.9%), and 103 (32.0%) patients, respectively. On histologic examination, the mean tumor size was 4.72 mm with 253 (78.6%) patients in the submucosa and 3 (0.9%) in the muscularis propria. LVI was positive in 11 (3.4%) patients and could not evaluated in 20 (6.2%); resection margin status revealed a positive margin in 57 (17.7%) and an indeterminate margin in 19 (5.9%). The median follow-up period was 40.49 months.

**Table 1:** Demographic and clinical characteristics of patients.

	Total (N = 322)	Risk negative (N = 180)	Risk indeterminate (N = 63)	Risk positive (N = 79)	*P*
***Characteristics of the patients***					
Age at diagnosis (year, mean (SD))	47.67 ± 11.47	46.80 ± 11.14	47.95 ± 12.67	48.62 ± 11.25	0.469
Gender (%)					0.143
Male	207 (64.3)	113 (62.8)	47 (74.6)	47 (59.5)	
Female	115 (35.7)	67 (37.2)	16 (25.4)	32 (40.5)	
How to be diagnosed (%)					0.041
Resected as NET	190 (59.0)	102 (56.7)	46 (73.0)	42 (53.2)	
Diagnosed via biopsy	102 (31.7)	64 (35.6)	13 (20.6)	25 (31.6)	
Resected as Polyps	30 (9.3)	14 (7.8)	4 (6.3)	12 (15.2)	
EUS (%)	44 (13.7)	20 (11.1)	6 (9.5)	18 (22.8)	0.024
Pelvic MRI (%)	15 (4.7)	8 (4.4)	2 (3.2)	5 (6.3)	0.661
Abdominal imaging (yes, %)					
Abdominopelvic CT	299 (92.9)	170 (94.4)	55 (87.3)	74 (93.7)	0.158
Abdominal US	40 (12.4)	19 (10.6)	9 (14.3)	12 (15.2)	0.513
Both	30 (9.3)	13 (7.2)	7 (11.1)	10 (12.7)	0.33
Not performed	13 (4.0)	4 (2.2)	6 (9.5)	3 (3.8)	0.04
Mode of treatment (%)					0.012
conventional EMR	97 (30.1)	44 (24.4)	21 (33.3)	32 (40.5)	
modified EMR	122 (37.9)	81 (45.0)	23 (36.5)	18 (22.8)	
ESD	103 (32.0)	55 (30.6)	19 (30.2)	29 (36.7)	
***Characteristics at histologic evaluation***					
Pathologic tumor size (mm, mean (SD))	4.72 ± 2.44	4.14 ± 1.90	4.45 ± 1.76	6.24 ± 3.24	<0.001
Group according to tumor size (%)					<0.001
<1 cm	301 (93.5)	180 (100.0)	63 (100.0)	58 (73.4)	
≥1 cm	21 (6.5)	0 (0.0)	0 (0.0)	21 (26.6)	
Tumor depth (%)					<0.001
Limited to mucosa	4 (1.2)	3 (1.7)	0 (0.0)	1 (1.3)	
Submucosa	253 (78.6)	177 (98.3)	13 (20.6)	63 (79.7)	
Muscularis propria	3 (0.9)	0 (0.0)	0 (0.0)	3 (3.8)	
Indeterminate	62 (19.3)	0 (0.0)	50 (79.4)	12 (15.2)	
Lymphovascular invasion (%)					<0.001
Negative	291 (90.4)	180 (100.0)	57 (90.5)	54 (68.4)	
Positive	11 (3.4)	0 (0.0)	0 (0.0)	11 (13.9)	
Indeterminate	20 (6.2)	0 (0.0)	6 (9.5)	14 (17.7)	
Resection margin (%)					<0.001
Negative	246 (76.4)	180 (100.0)	46 (73.0)	20 (25.3)	
Positive	57 (17.7)	0 (0.0)	0 (0.0)	57 (72.2)	
Indeterminate	19 (5.9)	0 (0.0)	17 (27.0)	2 (2.5)	
Follow-up period (months mean (SD), range)	40.49 ± 23.56 (4.27–102.7)	37.29 ± 22.90 (4.27–96.0)	39.93 ± 21.97 (8.0–89.6)	48.21 ± 24.98 (5.0–102.7)	0.002

NET, neuroendocrine tumor; CT, computed tomography; US, ultrasonography; EUS, endoscopic ultrasound; EMR, endoscopic mucosal resection;

ESD, endoscopic submucosal dissection; SD, standard deviation.

When comparing the baseline characteristics of patients according to the presence of risk factors determining curative resection, we categorized patients into risk negative, indeterminate, and positive groups according to known risk factors^11,14^ (size ≥1 cm, LVI, muscularis propria invasion, and positive resection margins). We defined the risk indeterminate and positive groups as non-curative resection. The rectal NETs initially resected as polyps and treated with conventional EMR were observed more frequently in the non-curative group (*P* = 0.041, and *P* = 0.012) (Table 1).

### Treatment outcomes of endoscopically resected rectal NETs

All rectal NETs were resected en bloc; procedure-related complications occurred in 11 patients (bleeding in 7 and suspected perforation in 4, 3.4%) all of whom were successfully treated with endoscopic methods (argon plasma coagulation and/or clips). The rate of complete resection and curative resection was 76.4% (246/322) and 55.9% (180/322), respectively. The results are summarized in Table 2.

**Table 2:** Outcomes of endoscopic resection of rectal NETs.

	Total (N = 322)	Risk negative (N = 180)	Risk indeterminate (N = 63)	Risk positive (N = 79)	*P*
En bloc resection (%)					
Yes	322 (100)	180 (100.0)	63 (100.0)	79 (100.0)	
No	0	0 (0.0)	0 (0.0)	0 (0.0)	
Complete resection (%)					<0.001
Complete (%)	246 (76.4)	180 (100.0)	46 (73.0)	20 (25.3)	
Incomplete (%)	57 (17.7)	0 (0.0)	0 (0.0)	57 (72.2)	
Indeterminate	19 (5.9)	0 (0.0)	17 (27.0)	2 (2.5)	
Complication					0.293
No	311 (96.6)	176 (97.8)	59 (93.7)	76 (96.2)	
Yes	11 (3.4)	4 (2.2)	4 (6.3)	3 (3.8)	
Surveillance frequency					
Endoscopic follow up	1.64 ± 0.92 (0–8)	1.58 ± 0.80	1.44 ± 0.69	1.94 ± 1.20	0.004
Imaging follow up	2.89 ± 2.49 (0–17)	2.51 ± 1.73	2.81 ± 2.61	3.65 ± 3.36	0.012

NET, neuroendocrine tumor.

Among the patients with endoscopically resected rectal NETs, a total of 44 patients received subsequent additive salvage treatment. As shown in Table 3, the most common reason to perform additional salvage treatment was a positive/indeterminate resection margin (38 patients, 86.4%), followed by a positive/indeterminate LVI (15, 34.1%), muscularis propria invasion (6, 13.6%) and size more than 1 cm (6, 13.6%). Of them, 11 (25.0%) patients underwent radical surgery, whereas others (75.0%) underwent local treatment including local excision in 11 (25.0%) patients, modified EMR in 12 (27.3%), coagulation in 6 (13.6%), and ESD in 4 (9.1%). Remnant tumor was found in 1 patient who was treated with subsequent ESD after initial EMR-C and lymph node metastasis was observed in 6 patients among 11 underwent radical surgery.

**Table 3:** Additional treatment after initial endoscopic resection of rectal NETs.

n = 44	n (%)
Reasons for additional treatment	
≥1 cm	6 (13.6)
Resection margin (Positive/Indeterminate)	38 (86.4)
Lymphovascular invasion (Positive/Indeterminate)	15 (34.1)
Muscularis propria invasion/Indeterminate	6 (13.6)
Treatment method	
ESD	4 (9.1)
modified EMR	12 (27.3)
Coagulation	6 (13.6)
Local excision (TAE/TEO)	11 (25.0)
Radical surgery	11 (25.0)
Treatment method	
Local	33 (75.0)
Radical	11 (25.0)
Residual tumor	1 (2.3)
LN metastasis	6 (13.6)

NET, neuroendocrine tumor; EMR, endoscopic mucosal resection.

ESD, endoscopic submucosal dissection; TAE, transanal excision.

TEO, transanal endoscopic operation; LN, lymph node.

### Factors associated with additional treatment after initial endoscopic resection

Notably, after ER, only 44 of the 142 patients (31.0%) who did not meet the criteria for curative resection received additional salvage treatment, whereas 98 did not because of either patient refusal or medical reasons. Therefore, we assessed the factors affecting the decision to perform additional salvage treatment after initial ER of rectal NETs (Table 4). Rectal NETs that were initially resected as polyps and then diagnosed and treated with conventional EMR were detected more frequently in the additional salvage treatment group (*P* < 0.001, and *P* = 0.006, respectively). In addition, there were more patients with positive or indeterminate LVI and resection margins after initial ER in the additional treatment group (*P* < 0.001, and *P* < 0.001, respectively)

**Table 4:** Risk factors for additional treatment after initial endoscopic resection.

	Univariate			Multivariate	
	Endoscopy only, N = 278	Endoscopy + additional Tx, N = 44	*P*	OR (95% CI)	*P*
Age at diagnosis (year, mean (SD))	47.33 ± 11.49	48.35 ± 11.41	0.582		
Gender (%)			0.358		
Male	176 (63.3)	31 (70.5)			
Female	102 (36.7)	13 (29.5)			
How to be diagnosed (%)			<0.001		
Resected as NET	169 (60.8)	21 (47.7)		0.040 (0.007–0.250)	0.001
Diagnosed via biopsy	93 (33.5)	9 (20.5)		0.096 (0.022–0.417)	0.002
Resected as Polyps	16 (5.8)	14 (31.8)		ref	
Mode of treatment (%)			0.006		
conventional EMR	75 (27.0)	22 (50.0)		ref	
modified EMR	112 (40.3)	10 (22.7)		1.304 (0.346–4.919)	0.695
ESD	91 (32.7)	12 (27.3)		1.124 (0.326–3.871)	0.853
Pathologic tumor size (mm, mean (SD), range)	4.63 ± 2.40	5.31 ± 2.62	0.085		
Group according to tumor size (%)			0.051		
<1 cm	263 (94.6)	38 (86.4)		ref	
≥1 cm	15 (5.4)	6 (13.6)		3.035 (0.605–15.235)	0.177
Tumor depth (%)			0.054		
Limited to mucosa	3 (1.1)	1 (2.3)		ref	
Submucosa	218 (78.4)	35 (79.5)		0.499 (0.028–8.811)	0.635
Muscularis propria	1 (0.4)	2 (4.5)		1.401 (0.030–65.233)	0.863
Indeterminate	56 (20.1)	6 (13.6)		0.453 (0.021–9.991)	0.616
Lymphovascular invasion (%)			<0.001		
Negative	262 (94.2)	29 (65.9)		ref	
Positive	5 (1.8)	6 (13.6)		61.971 (9.138–420.248)	<0.001
Indeterminate	11 (4.0)	9 (20.5)		2.026 (0.516–7.971)	0.312
Resection margin (%)			<0.001		
Negative	240 (86.3)	6 (13.6)		ref	
Positive	24 (8.6)	33 (75.0)		75.993 (20.943–275.751)	<0.001
Indeterminate	14 (5.0)	5 (11.4)		13.203 (2.686–64.902)	0.001

NET, neuroendocrine tumor; EMR, endoscopic mucosal resection; ESD, endoscopic submucosal dissection; SD, standard deviation.

In multivariate analysis, lesions diagnosed via biopsies (OR, 0.096; *P* = 0.002) or suspected as NETs initially (OR, 0.04; *P* = 0.001) were less likely to undergo additional treatment. Whereas, positive LVI (OR 61.971; P < 0.001), positive (OR 75.993; *P* < 0.001), or indeterminate (OR 13.203; *P* = 0.001) resection margins were associated with the decision to perform additional treatment.

### Clinical outcomes during follow-up

Surveillance endoscopy was performed in 277 patients (86.0%), and abdominal imaging was performed in 220 patients (68.3%) with endoscopically resected rectal NETs. The mean frequency of endoscopy and abdominal imaging was 1.64 ± 0.92 (range, 0–8) and 2.89 ± 2.49 (range, 0–17), respectively. Both surveillance endoscopy and abdominal imaging were done more frequently in the non-curative group as shown in Table 2 (P = 0.004, and *P* = 0.012, respectively).

Lymph node metastasis was detected in 6 of 11 patients undergoing radical surgery after initial ER. The characteristics of patients with LN metastasis after ER are listed in Table 5. Among them, 5 patients received chemotherapy followed by radical surgery. No patients including those with additional salvage treatment experienced local or metastasis tumor recurrence during the follow-up period.

**Table 5:** Clinicopathologic characteristics of patients who developed LN metastasis of rectal NETs.

No.	Sex	Age	Tumor size (mm)	Depth of invasion	LVI	Resection margin	Initial Treatment	Additional Treatment	F/U duration (mo)	Outcome
1	M	35	10	Submucosa	Positive	Positive	ESD	LAR	42	No recur
2	M	36	10	Submucosa	Indeterminate	Positive	EMR	LAR	90	No recur
3	M	60	10	Muscularis propria	Indeterminate	Positive	EMR-C	LAR	100	No recur
4	F	57	10	Submucosa	Negative	Positive	EMR	LAR	77	No recur
5	M	41	7	Submucosa	Indeterminate	Positive	ESD	LAR	72	No recur
6	M	69	13	Muscularis propria	Negative	Positive	ESD	LAR	63	No recur

LVI, lymphovascular invasion; EMR, endoscopic mucosal resection; ESD, endoscopic submucosal dissection; NET, neuroendocrine tumor; EMR-C, EMR with a cap; LAR, low anterior resection.

## Discussion

In this study, we evaluated the factors determining additional salvage treatments after ER of rectal NETs and long-term clinical outcomes according to additional treatments of endoscopically resected rectal NETs. Recently, the detection of rectal NETs is increasing with the widespread use of screening colonoscopy^15^. Therefore, most rectal NETs are small and localized^16^. Although patients with rectal NETs have a good prognosis, patients with liver metastasis or distant lymph node metastasis showed a poor prognosis^3,17^. Rectal NETs that had a tumor size <1 cm without muscularis propria invasion and without lymph node or distant metastasis could be treated with local excision or ER^18,19^. According to the current guidelines, lesions considered to be histologically non-curatively resected after ER must undergo additional salvage treatments^11^. However, the majority of patients with non-curative resection did not undergo additional salvage treatments^20^. The reason for patients not receiving additional treatments is that positive resection margins are not always predictive factors of remnant tumor, recurrence, or metastasis and that patients or clinicians decide to choose regular follow-up through colonoscopy and biopsy rather than salvage treatments. However, to our knowledge, there are no large-scale studies that have evaluated the long-term outcomes of rectal NETs treated with only ER compared with those treated with additional salvage treatments after ER for non-curatively resected rectal NETs. In addition, there are no recommended criteria or consensus regarding treatment strategies after non-curative resection of rectal NETs that have positive resection margin, tumor size ≥1 cm, and LVI. Therefore, in this study, we focused on the true prognosis of endoscopically resected rectal NETs according to additional treatments after non-curative resection and clarified the factors that determined the salvage treatments.

In this study, only 44 of the 142 patients (31.0%) who did not meet the criteria for curative resection received additional salvage treatment. The factors determining additional treatments are lesions resected as polyps, positive LVI, and positive/indeterminate resection margins. The majority of patients (38/44, 86.4%) were received additive salvage treatments because of the positive resection margin. A total of 11 patients underwent radical surgery and the others local treatments such as ER, coagulation or local excision. Approximately 50% of the patients who received additional treatments were treated endoscopically and the modified EMR was most used for salvage treatments. Because lymph node dissection can be performed in radical surgery, we investigated the clinicopathologic factors of patients who underwent radical surgery. Most of them were found to have positive lymphovascular invasion, muscularis propria invasion, and grade 3 which are the risk factors of lymph node metastasis (Supplement Table 1). There are few studies about the superiority about the methods of additional salvage treatments following non-curative resection for rectal NETs. However, no local recurrence and distant metastasis were observed during follow-up in all 322 patients who underwent initially ER for rectal NETs. Therefore, the endoscopically resected rectal NETs showed a favorable prognosis regardless of additional salvage treatments in case of non-curative resection.

However, it should not be overlooked that 6 of 11 patients who underwent radical surgery after ER showed LN metastasis. Although rectal NETs are known to have a good prognosis with 5-year survival rate of 74–88%^4^, patients with high risk of LN metastasis need to undergo radical surgery with LN dissection. Our results are in agreement with those of a previous study by Park *et al*.^21^ that evaluated the clinical outcomes of patients with rectal NET (<2 cm); among 304 patients treated with ER, 3 of the 10 patients who underwent additional surgery after initial ER had LN metastasis, all of whom did not experience tumor recurrence during the follow-up period. The risk factors for LN metastasis were tumor size (>14 mm), increased mitotic rate, and LVI. Therefore, future studies are necessary to identify the patients who need to undergo radical surgery by revealing the risk factors for LN metastasis.

There are a few studies that have investigated the clinical outcomes of rectal NETs with only ER^20,22,23^. In a previous study, Moon *et al*. reported that local recurrence occurred in 3 (0.74%) patients among 407 patients who underwent ER of rectal NETs^20^. In that study, only 14 patients received salvage treatments after ER, although there were more patients who showed non-curative resection. This means that similar to our study, not all patients with non-curative resection underwent salvage treatments. However, in our data, the proportion of patients who received additional salvage treatments was higher than those in the study by Moon and there was no local recurrence (0% vs. 0.7%) during follow-up. We cannot conclude that additional salvage treatments lowered the local recurrence rates. Therefore, further studies are needed about the role of additional salvage treatments for lowering the local recurrence rate. However, both studies had no patients who experienced distant metastasis or disease-related morbidity.

According to current recommended guidelines, the National Comprehensive Cancer Network guidelines and the European Neuroendocrine Tumors Society consensus guidelines suggest that no regular follow-up is usually required for rectal NETs <1 cm^10,24^. In this study, neither local recurrence and nor distant metastasis was observed in any cases during follow-up. However, the surveillance frequency with endoscopy and imaging studies were higher in the risk indeterminate and positive group than in the risk negative group. Therefore, it is necessary to evaluate how often follow-up should be done after non-curative resection of rectal NETs.

This study has several limitations. First, this is a retrospective study. Second, the prognostic factors such as mitotic, Ki-67 indices, and tumor differentiation were not evaluated in all patients. Third, we only included patients who underwent ER for rectal NETs and the patients treated initially with surgery were excluded. The prognosis might be different if these patients had been included.

Despite these limitations, to the best of our knowledge, ours is the first study to evaluate the factors determining additional salvage treatments after ER of rectal NETs, additional treatment modalities in case of non-curative resection, and long-term clinical outcomes according to additional salvage treatments of endoscopically resected rectal NETs. It would also be helpful for endoscopists to determine treatment strategies and predict prognosis of patients with rectal NETs treated endoscopically in clinical practice.

In conclusion, the long-term outcome after ER for rectal NETs was excellent. Positive LVI, positive or indeterminate resection margins, and initial non-suspected NETs were factors for determining additional treatments. However, no local recurrence and distant metastasis were seen regardless of whether patients received additional salvage treatments after non-curative resection. Therefore, the rectal NETs which underwent initially ER might show a favorable prognosis in patients with risk indeterminate or positive group.

## Method

### Patients

**Figure 1:**
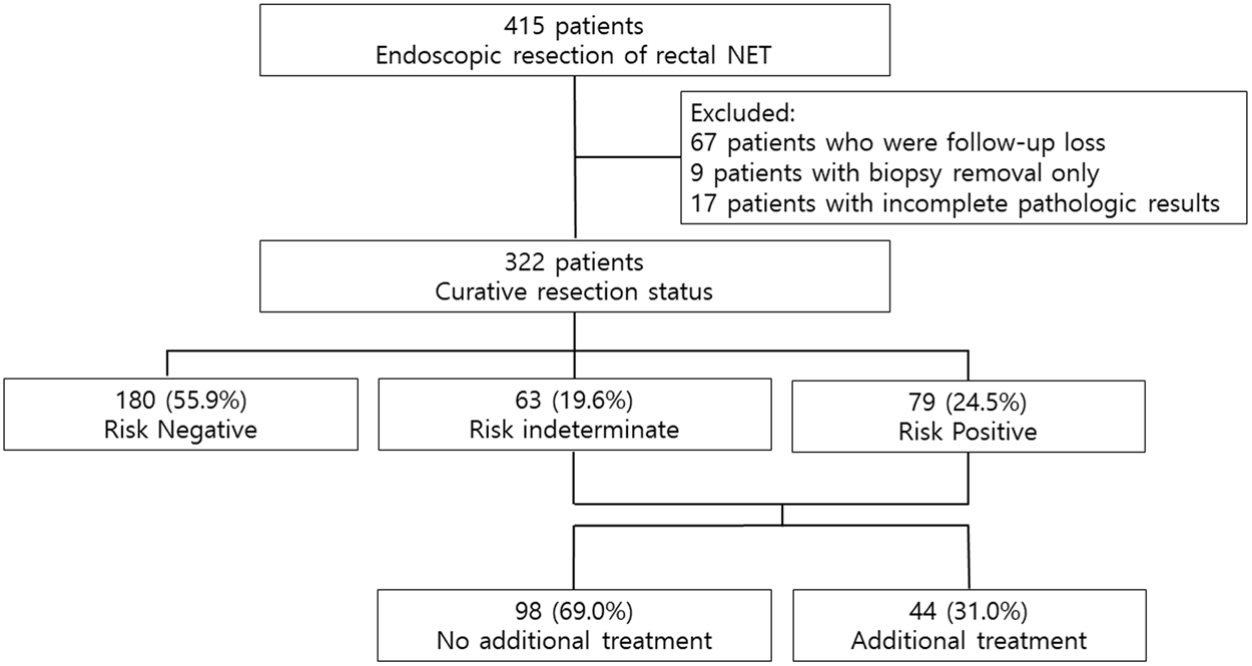
Schematic flow chart of the patients in this study. CT, computed tomography; US, ultrasonography; NET, neuroendocrine tumor.

We retrospectively reviewed the clinical data on patients pathologically diagnosed with rectal NETs who underwent ER at the Severance and Gangnam Severance Hospitals, Yonsei University College of Medicine, Seoul, Korea, between January 2005 and December 2016. The patients were evaluated using abdominopelvic computed tomography (CT) or ultrasonography (US) before undergoing ER to exclude metastases to the regional lymph nodes and/or distant organs. A total of 415 patients with rectal NETs were initially reviewed in this study. Of these, 93 patients were excluded for the following reasons: lost to follow-up (n = 67), biopsy removal only (n = 9), and incomplete pathologic results (n = 17). Finally, 322 patients were included in this study (Fig. 1).

Informed consent was obtained from all patients before the procedures. This study was approved by the Institutional Review Board of Yonsei University College of Medicine and was conducted in accordance with the ethical principles of the Declaration of Helsinki.

### Endoscopic procedure

The optimal ER technique for rectal NETs was decided by experienced endoscopists, depending on the size and endoscopic appearance of the tumor. The resection methods for rectal NETs were classified as follows: conventional endoscopic mucosal resection (EMR) including polypectomy; modified EMR including cap-assisted EMR, ligation-assisted EMR, and precut EMR; and endoscopic submucosal dissection (ESD). En bloc resection was defined as resection of the entire lesion in a single piece. Endoscopic complete resection was determined as en bloc resection with the resected tissue having negative margins. We also evaluated procedure-related complications, if any.

### Histopathologic examination

Resected specimens were evaluated histologically using light microscopy to determine tumor size, tumor depth, LVI, and lateral and vertical resection margin involvement. Immunohistochemical staining for neuron-specific enolase, synaptophysin, and chromogranin A was performed to support the diagnosis. Resection margin was classified as positive, negative, or indeterminate. An indeterminate resection margin was defined if an endoscopically removed specimen was in fragments or the margins could not be adequately assessed. Curative resection was determined as endoscopic complete resection of tumor less than 1 cm in size without LVI and whose depth was within the submucosa.

In addition, we classified patients into 3 risk groups according to the curative resection status to evaluate the clinical outcomes. The patients with no risk factors were classified as risk negative and those with at least one risk factor were classified as risk positive. In other words, the patients are indicated as a risk negative group when all of the following condition are fulfilled: en-bloc resection, tumor size <1 cm, no LVI, confined submucosa, and negative resection margin. And, the patients are regarded as a high risk group if the tumors have any of the followings: size ≥1 cm, LVI, muscularis propria invasion, or positive resection margins. Therefore, the remaining patients were defined as risk indeterminate such as indeterminate depth, LVI, and resection margin.

### Additional treatment

Among the patients who did not meet the criteria for curative resection, some received additional treatments for non-curative lesions. The reasons for additional treatments, treatment methods, and pathologic findings (residual tumor or LN metastasis status) were also assessed in this study. The treatment methods were classified into modified EMR, ESD, local excision (transanal endoscopic operation [TEO], transanal excision [TAE]), or radical surgery with lymph node dissection (low anterior resection [LAR]).

### Statistical analysis

Categorical variables are presented as numbers with percentages and were compared using the chi-square or Fisher’s exact test. Continuous variables are presented as means ± standard deviations and were compared with the aid of Student’s *t*-test or one-way ANOVA test. To identify the factors affecting the decision to perform additional treatment after the initial ER of rectal NETs, we performed both univariate and multivariate logistic regression analyses. A *P* value < 0.05 was considered to reflect statistical significance. All statistical analysis was performed using SPSS software, version 23.0 for Windows (SPSS, Chicago, Illinois, USA).

## Supplementary information

Supplementary Table 1
