# Supplementary material for: Long-term outcomes according to additional treatments after endoscopic resection for rectal small neuroendocrine tumors
Source: Sci Rep. 2019 Mar 20;9:4911. doi: 10.1038/s41598-019-40668-6 (PMC6426846; doi:10.1038/s41598-019-40668-6)
Supplement: Supplementary file 1 — Supplementary Table 1 [file 41598_2019_40668_MOESM1_ESM.docx]

Title page

Original article

Long-term outcomes according to additional treatments after endoscopic resection

for rectal small neuroendocrine tumors

Jae Hwang Cha, MD^4^, Da Hyun Jung, MD^1^, Jie-Hyun Kim, MD, PhD^1^,

Young Hoon Youn, MD, PhD^1^, Hyojin Park, MD, PhD^1^, Jae Jun Park, MD, PhD^1^, Yoo Jin Um, MD ^1^,

Soo Jung Park, MD, PhD^2^, Jae Hee Cheon, MD, PhD^2^, Tae Il Kim, MD, PhD^2^,

Won Ho Kim, MD, PhD^2^, Hyun Jung Lee, MD, PhD^2,3^

^1^Department of Internal Medicine, Gangnam Severance Hospital, Yonsei University College of Medicine, Seoul, 06273, Korea

^2^Department of Internal Medicine, Institute of Gastroenterology, Yonsei University College of Medicine, Seoul,03722, Korea

^3^Department of Internal Medicine and Liver Research Institute, Seoul National University College of Medicine, Seoul, 03080, Korea

^4^Department of Internal Medicine, Dong-A University College of Medicine, Busan,49201, Korea

**Running head:** Long-term outcomes for rectal NETs

**Corresponding author**:

1. Da Hyun Jung, MD

**Address:** Department of Internal Medicine, Gangnam Severance Hospital, Yonsei University College of Medicine, 211 Eonjuro, Gangnam-gu, Seoul, Korea,06273

**Phone:** 82-2-2019-3310

**Fax:** 82-2-3463-3882

**E-mail:** [leah1004@yuhs.ac](mailto:leah1004@yuhs.ac)

2. Hyun Jung Lee, MD, PhD

**Address:** Department of Internal Medicine and Liver Research Institute, Seoul National University College of Medicine, 101 Daehak-ro, Jongno-gu, Seoul, Korea, 03080

**Phone:** 82-2-2072-3439

**Fax:** 82-2-762-9662

**E-mail:** [guswjd80@gmail.com](mailto:guswjd80@gmail.com)

Supplementary Table 1 Clinicopathologic characteristics of patients who received radical surgery after endoscopic resection of rectal NETs

| No. | Sex | Age | Tumor size (mm) | Depth of  invasion | LVI | Resection margin |
| --- | --- | --- | --- | --- | --- | --- |
| 1 | M | 35 | 10 | Submucosa | Positive | Positive |
| 2 | M | 36 | 10 | Submucosa | Indeterminate | Positive |
| 3 | M | 60 | 10 | Muscularis propria | Indeterminate | Positive |
| 4 | F | 57 | 10 | Submucosa | Negative | Positive |
| 5 | M | 41 | 7 | Submucosa | Indeterminate | Positive |
| 6 | M | 69 | 13 | Muscularis propria | Negative | Positive |
| 7 | M | 56 | 7 | Submucosa | Positive | Negative |
| 8 | F | 55 | 5 | Submucosa | Positive | Indeterminate |
| 9 | F | 47 | 10 | Submucosa | Indeterminate | Positive |
| 10 | M | 68 | 3 | Submucosa | Positive | Negative |
| 11 | F | 49 | 5 | Submucosa | Positive | Negative |
